# Lead Toxicity Masquerading as Autoimmune Haemolytic Anaemia: A Diagnostic Pitfall in Unexplained Anaemia

**DOI:** 10.7759/cureus.102622

**Published:** 2026-01-30

**Authors:** John Ee Chew, Nathan Klose

**Affiliations:** 1 Gastroenterology and Hepatology, University of Queensland, Queensland, AUS; 2 Hematology, Greenslopes Private Hospital, Queensland, AUS

**Keywords:** anaemia, basophilic stippling, blood smear, chelation therapy, dietary supplements, haemolytic, lead poisoning, pappenheimer bodies

## Abstract

Lead toxicity is an uncommon but clinically important cause of unexplained anaemia. Its heterogeneous haematological manifestations can mimic autoimmune haemolytic anaemia, resulting in diagnostic uncertainty and delayed recognition.

A 49-year-old woman presented with a three-week history of worsening abdominal pain and lethargy. She had a background of inclusion body myositis treated with long-term intravenous immunoglobulin and recent initiation of mycophenolate mofetil. Initial investigations demonstrated severe anaemia with reticulocytosis but minimal biochemical evidence of haemolysis. A weakly positive direct antiglobulin test (IgG 1+, C3d negative) raised suspicion for autoimmune haemolysis. The initial peripheral blood film was reported as showing marked red cell agglutination without additional abnormalities. Progressive anaemia prompted extensive investigations, including infectious, immunological, and haematological workups, all of which were unremarkable. Bone marrow aspirate demonstrated atypical plasma cells, prompting re-examination of the peripheral blood film, which revealed Pappenheimer bodies and basophilic stippling. Further history identified ingestion of an unlabelled health supplement, and toxicology testing confirmed markedly elevated blood lead levels. Chelation therapy with succimer led to clinical and haematological improvement. Public health investigation subsequently confirmed the supplement as the source of lead and mercury exposure.

This case highlights lead toxicity as an important but under-recognised mimic of autoimmune haemolytic anaemia. Careful review of peripheral blood morphology and a thorough exposure history are essential when evaluating unexplained anaemia with an inconclusive haemolysis workup.

## Introduction

Lead poisoning remains a rare but serious condition and can originate from traditional remedies, including Ayurvedic medicine. These supplements sometimes contain toxic heavy metals, intentionally or via contamination, posing risks of haematologic, renal, and neurologic toxicity [1,2]. Among its haematological manifestations, lead toxicity may present with anaemia, reticulocytosis, and basophilic stippling and can mimic haemolytic disorders, particularly autoimmune haemolytic anaemia (AIHA), resulting in diagnostic confusion [2-4].

Mechanistically, lead interferes with key enzymes in the haem biosynthesis pathway, including δ-aminolevulinic acid dehydratase and ferrochelatase, resulting in impaired haem production, ineffective erythropoiesis, and characteristic morphological findings such as coarse basophilic stippling and sideroblastic features [4,5]. Inhibition of pyrimidine-5′-nucleotidase further contributes to the accumulation of ribosomal RNA in red cells, producing the stippling seen on peripheral blood smears [6]. These changes, along with red cell destruction and marrow erythroid hyperplasia, can closely resemble immune-mediated haemolysis.

Diagnostic challenges arise when partial or misleading findings, such as a weakly positive direct antiglobulin test or red cell agglutination, suggest AIHA, particularly in patients receiving immunosuppressive therapy. For example, intravenous immunoglobulin (IVIG) has been associated with false-positive Coombs tests, further complicating interpretation [7]. Without prominent biochemical markers of haemolysis, clinicians may pursue extensive immunologic and haematologic evaluations while overlooking toxic or environmental causes.

Here, we report a diagnostically challenging case of lead toxicity masquerading as autoimmune haemolytic anaemia in a patient receiving long-term immunosuppressive therapy. This case underscores the importance of thorough exposure history, including use of unregulated supplements, and careful peripheral blood smear review in evaluating unexplained anaemia. Although lead toxicity from traditional remedies has been reported, its presentation as an AIHA mimic remains under-recognised [3,4,8]. Our case highlights a key diagnostic pitfall and reinforces the need for a structured and vigilant approach in similar clinical scenarios.

## Case presentation

A 49-year-old woman presented with a three-week history of worsening abdominal pain and progressive lethargy. Her medical history included inclusion body myositis managed with long-term intravenous immunoglobulin (IVIG) and recent initiation of mycophenolate mofetil. There was no recent infection, gastrointestinal bleeding, or known toxin exposure reported initially.

**Figure 1:**
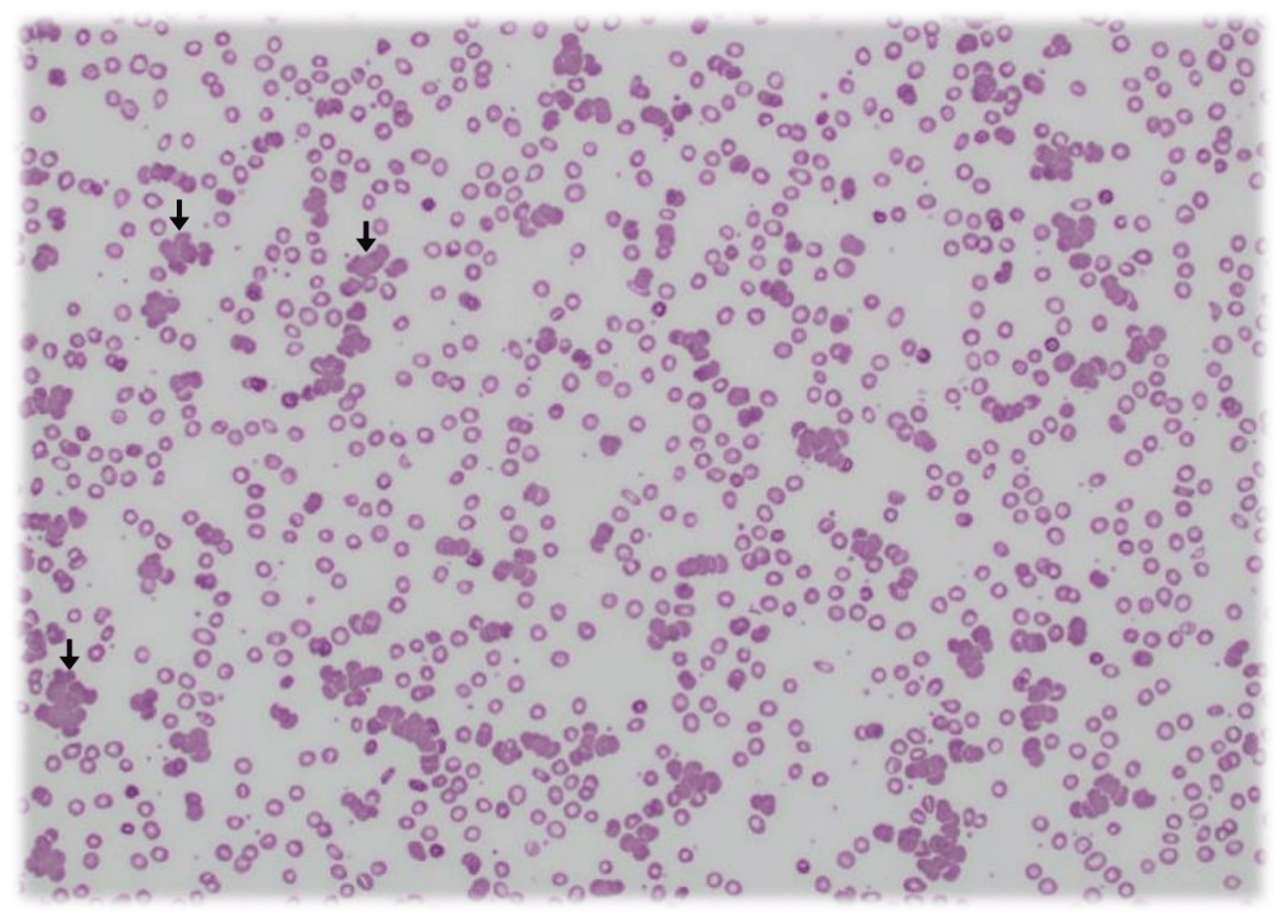
Peripheral blood smear at initial presentation Marked red blood cell agglutination (arrows), which led to a presumptive diagnosis of autoimmune haemolytic anaemia. This morphological finding, in conjunction with a weakly positive direct antiglobulin test, supported the initial working diagnosis.

Admission laboratory results revealed marked normocytic anaemia with haemoglobin of 72 g/L and reticulocytosis of 190 × 10⁹/L (Table 1), suggestive of an appropriate marrow response (Appendix). However, markers of biochemical haemolysis were only modestly abnormal (Table 2): total bilirubin was 29 µmol/L (reference <16 µmol/L), lactate dehydrogenase (LDH) was mildly elevated at 339 U/L (reference 120-250 U/L), and haptoglobin was low-normal at 0.20 g/L (reference 0.16-2.00 g/L). A direct antiglobulin test (DAT) was weakly positive for IgG (1+) but negative for C3d, raising initial suspicion for warm autoimmune haemolytic anaemia (AIHA). The initial peripheral blood film reported by the laboratory haematologist described marked red cell agglutination without other abnormalities (Figure 1).

**Table 1:** Initial haematological parameters Initial haematological parameters at presentation showed normocytic anaemia with marked reticulocytosis, indicating an active erythroid response. Despite this, the diagnosis remained uncertain until morphological features on the blood film prompted investigation for lead toxicity. Note: Arrows indicate directional change. Marked anaemia with reticulocytosis suggested a regenerative response. Further investigation was required due to the absence of classical haemolytic features and subsequent identification of basophilic stippling.

Parameter	04/04/22	13/07/22 (Admission)	Reference Range	Units
Haemoglobin	127	72 ↓	115–165	g/L
Haematocrit	0.38	0.22 ↓	0.35–0.47	-
Red Cell Count (RCC)	4.1	2.4 ↓	3.9–5.6	10¹²/L
Reticulocytes	-	190 ↑	25–120	10⁹/L
Mean Cell Volume (MCV)	93	93	80–100	fL
White Cell Count (WCC)	4.5	4.9	3.5–12.0	10⁹/L
Platelets	183	214	150–400	10⁹/L

**Table 2:** Biochemical and haematological investigations at presentation Laboratory findings demonstrating anaemia with appropriate marrow response and mild biochemical evidence of haemolysis. Elevated LDH (339 U/L), indirect hyperbilirubinaemia (29 µmol/L), and low-normal haptoglobin (0.20 g/L) were noted. The direct Coombs test was weakly positive (anti-IgG 1+, anti-C3d non-reactive), raising suspicion for autoimmune haemolytic anaemia. Iron studies showed normal ferritin with low transferrin and total iron-binding capacity (TIBC), but increased saturation. Folate and B12 levels were within normal range. These findings contributed to diagnostic uncertainty and prompted further evaluation. CRP: C-reactive protein, AST: Aspartate aminotransferase, ALT: Alanine transaminase, LDH: Lactate dehydrogenase, CK: Creatine kinase

Test Name	Result	Units	Reference Interval
Iron	24	umol/L	5 - 30
Transferrin	1.8 L	g/L	1.9 - 3.1
TIBC	45 L	umol/L	47 - 77
Saturation	53 H	%	20 - 45
Ferritin	165	ug/L	30 - 300
CRP	<0.4	mg/L	<5
Vitamin B12	375	pmol/L	>150
Active B12	>128	pmol/L	>35
Folate (Serum)	39	nmol/L	>7.0
Bilirubin	29 H	umol/L	<16
Alk Phos	42	U/L	20 - 105
AST	56 H	U/L	10 - 35
ALT	68 H	U/L	5 - 30
Gamma GT	13	U/L	5 - 35
LDH	339 H	U/L	120 - 250
Cholesterol	4.5	mmol/L	<5.6
CK	481 H	U/L	30 - 150
Magnesium	0.82	mmol/L	0.70 - 1.10
Haemolysis Index	4		<40
Haptoglobin	0.20	g/L	0.16 - 2.00
Direct Coombs Test	Positive		
Anti-IgG	1+		(Scale 1 to 4)
Anti-C3d	Non Reactive		(Scale 1 to 4)
Antibody Screen	Negative		
Blood Group	A Rh(D) Positive		

**Figure 2:**
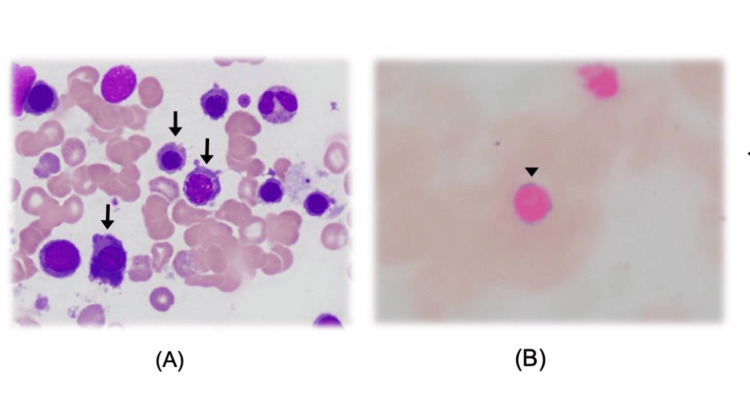
Bone marrow aspirate film A) Peripheral blood smear (May-Grünwald-Giemsa stain) demonstrating dyserythropoietic features, including coarse basophilic stippling (arrows), within erythrocytes. Basophilic stippling represents precipitated ribosomal RNA caused by pyrimidine-5′-nucleotidase inhibition—a hallmark of lead toxicity. (B) Iron stain (Prussian Blue) highlighting a ring sideroblast (arrowhead), indicative of mitochondrial iron loading due to impaired heme synthesis. This finding reflects ineffective erythropoiesis, consistent with lead-induced disruption of ferrochelatase activity in the heme biosynthetic pathway. The combination of basophilic stippling and ring sideroblasts provided key morphological evidence supporting the diagnosis of lead poisoning, which had initially been overlooked.

Given the diagnostic uncertainty, an extensive workup was undertaken, including viral serologies (EBV, Mycoplasma), autoimmune screens, cold agglutinin titres, and urinary haemosiderin, all of which were unremarkable (Table 3). Despite these investigations, the patient’s haemoglobin continued to decline, prompting a bone marrow aspirate. This revealed dyserythropoietic features and coarse basophilic stippling (Figure 2), which are characteristic of lead poisoning.

**Table 3:** Immunological, infectious, and cold agglutinin workup Expanded investigations undertaken to evaluate unexplained haemolytic anaemia. Serum protein electrophoresis and immunofixation revealed no monoclonal gammopathy. While immunoglobulin A was slightly reduced and free light chains (kappa and lambda) elevated, the kappa/lambda ratio remained normal. Infectious serologies for *Mycoplasma pneumoniae* and Epstein-Barr virus (EBV) showed negative IgM titres, with EBV NA IgG positivity consistent with past infection. Cold agglutinin testing demonstrated borderline elevation in patient cell agglutination at 4°C (titre 32; upper limit of normal), but no significant agglutination at higher temperatures, suggesting low thermal amplitude and limited clinical relevance. Urinary haemosiderin was not detected. Overall, these findings did not support an autoimmune or infectious haemolytic process, prompting reconsideration of alternative causes.

Test	Result	Units	Reference Interval
Albumin	40	g/L	33 - 46
Alpha 1	2	g/L	2 - 4
Alpha 2	4	g/L	4 - 9
Beta 1	3	g/L	2 - 6
Beta 2	1.5	g/L	2 - 6
Gamma	15	g/L	6 - 15
Total Protein	67	g/L	64 - 81
Immunofixation	No Monoclonal Immunoglobulin Detected		
Immunoglobulin G (IgG)	14.90	g/L	5.76 - 15.36
Immunoglobulin A (IgA)	1.12 L	g/L	1.24 - 4.16
Immunoglobulin M (IgM)	1.79	g/L	0.48 - 3.1
Kappa Free Light Chains	30 H	mg/L	7 - 22
Lambda Free Light Chains	37 H	mg/L	8 - 27
K/L Ratio	0.81		0.31 - 1.56

Test	Result	Method/Notes
Mycoplasma IgG (CLIA)	Negative	
Mycoplasma IgM (CLIA)	Negative	
EBV VCA IgM (CLIA)	Negative	
EBV NA IgG (CLIA)	Positive	Consistent with past infection

Test	Result	Units	Reference Range
Urinary Haemosiderin	Not Detected		
Cold Agglutinin Titre (4°C) - Patient Cells	32		0 - 32

**Figure 3:**
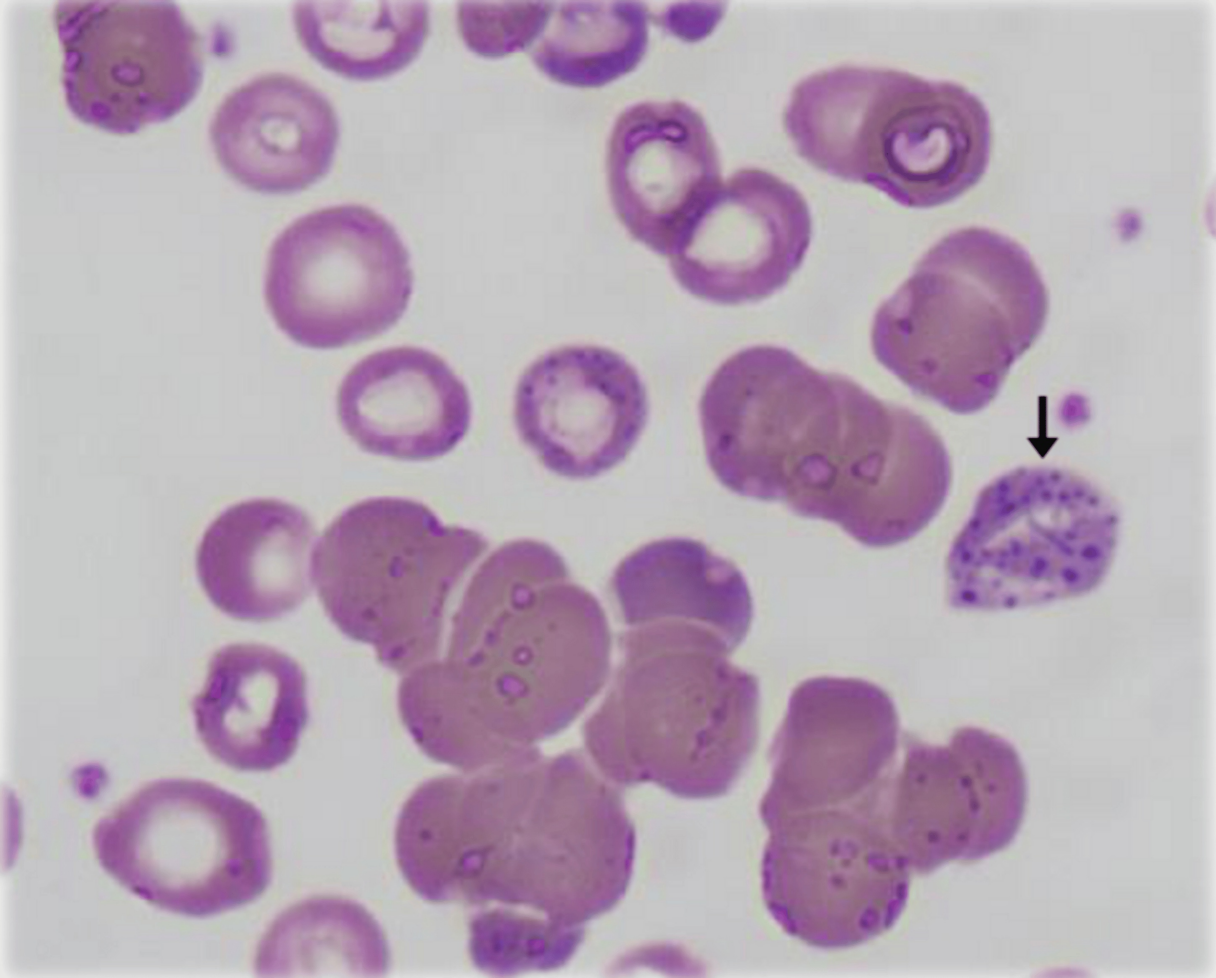
Re-examination of the initial peripheral blood film Coarse basophilic stippling (arrow) within erythrocytes. These features—initially overlooked—are classic morphological clues of lead toxicity and prompted confirmatory toxicology testing.

In view of the bone marrow findings, a re-examination of the initial peripheral smear was performed. On closer inspection, previously overlooked features, basophilic stippling, were identified (Figure 3), raising strong suspicion for heavy metal toxicity, particularly lead [3,4]. At this point, a targeted exposure history was revisited, during which the patient disclosed recent ingestion of an unlabelled herbal supplement provided by a friend.

Toxicology testing confirmed markedly elevated blood lead levels. The patient was promptly commenced on oral chelation therapy with succimer after consultation with a toxicology specialist. This led to progressive haematological recovery and symptomatic improvement (Tables 4, 5). The case was reported to public health authorities, who confirmed the supplement as the source of lead, arsenic, and mercury contamination and coordinated community-level mitigation.

**Table 4:** Serial haematological and biochemical parameters during admission The trend of haematological indices over six consecutive days, showing persistent normocytic anaemia (lowest Hb 64 g/L on 17/07/2022) with sustained reticulocytosis (peak 286 × 10⁹/L), indicating ongoing marrow compensation. Red cell count (RCC) and haematocrit remained low throughout. Total bilirubin, LDH, and transaminases were variably elevated, supporting a haemolytic process, though not definitive for autoimmune haemolysis. The nucleated red blood cell (NRBC) count remained mildly elevated. Notably, no clear spontaneous improvement was observed until diagnosis and chelation therapy were initiated. These findings illustrate the biochemical ambiguity of the case and reinforce the diagnostic challenge prior to identifying lead toxicity. MCV: Mean corpuscular volume, WCC: White cell count, ALP: Alkaline phosphatase, AST: Aspartate transferase, ALT: Alanine transaminase, GGT: Gamma-glutamyl transferase, LDH: Lactate dehydrogenase

Test	14/07/22	15/07/22	16/07/22	17/07/22	18/07/22	19/07/22	Reference	Units
Haemoglobin	72 L	77 L	72 L	64 L	75 L	66 L	(115 - 165)	g/L
Haematocrit	0.21 L	0.23 L	0.22 L	0.20 L	0.23 L	0.20 L	(0.35 - 0.47)	L/L
RCC	2.3 L	2.5 L	2.4 L	2.1 L	2.5 L	2.1 L	(3.9 - 5.6)	10^12/L
Reticulocytes	193 H	234 H		202 H		286 H	(25 - 120)	10^9/L
MCV	91	93	92	94	94	93	(80 - 100)	fL
WCC	5.0	5.2	6.2	5.2	5.6	7.1	(3.5 - 12.0)	10^9/L
Neutrophils	3.06	2.86	3.71	2.68	3.44	4.59	(1.5 - 8.0)	10^9/L
Lymphocytes	1.39	1.79	1.87	1.97	1.71	1.74	(1.0 - 4.0)	10^9/L
Monocytes	0.50	0.40	0.48	0.43	0.38	0.67	(0.0 - 0.9)	10^9/L
Eosinophils	0.05	0.10	0.13	0.09	0.10	0.13	(0.0 - 0.6)	10^9/L
Basophils	0.00	0.00	0.01	0.01	0.01	0.01	(0.0 - 0.15)	10^9/L
NRBC	1.0 H						(0)	10^9/L
Platelets	200	246	217	201	205		(150 - 400)	10^9/L
T Bilirubin	27 H	32 H	25 H	19 H	17 H	12	(<16)	umol/L
ALP	39	44	41	34	41	36	(20 - 105)	U/L
AST	45 H	46 H	47 H	41 H	52 H	48 H	(10 - 35)	U/L
ALT	58 H	55 H	54 H	47 H	60 H	55 H	(5 - 30)	U/L
GGT	13	15	16	13	15	15	(5 - 35)	U/L
LDH	273 H	337 H	302 H	243	284 H	258 H	(120 - 250)	U/L

**Table 5:** Blood lead levels and interpretation guidelines Guidelines for blood lead interpretation: 1. No workplace exposure: - Blood lead > 5 µg/dL (0.24 µmol/L): the source of exposure should be investigated and removed. - Chelation therapy (adults) is considered if blood lead is > 69.9 µg/dL (3.33 µmol/L) or if symptoms of high exposure are present.

Test	Result	Units	Reference Range
Lead-blood	3.76 H	umol/L	0 - 0.24
Lead-blood	77.8 H	ug/dL	0 - 5

## Discussion

Lead poisoning remains an uncommon but clinically important cause of unexplained anaemia and abdominal pain, and it can masquerade as autoimmune or microangiopathic haemolytic processes [2-4]. Lead interferes with several enzymes in the haem biosynthesis pathway (notably δ-aminolevulinic acid dehydratase and ferrochelatase), producing impaired haem synthesis, accumulation of erythroid precursors, and characteristic morphological changes such as coarse basophilic stippling and sideroblastic features [4,5].

Basophilic stippling, coarse, blue granular inclusions within erythrocytes on a Wright/Giemsa stain, results from the precipitation of ribosomal RNA caused by inhibition of pyrimidine-5′-nucleotidase and is a classic morphologic clue to lead [6]. Because basophilic stippling may be focal or subtle, it can be overlooked when more prominent or competing smear findings (for example, marked agglutination or numerous nucleated red cells) are present; careful review by an experienced haematologist or repeat slide review is therefore often decisive.

Traditional and herbal medicines, including some Ayurvedic preparations, are a well-documented source of lead and other heavy-metal exposures worldwide [1,5,7]. Surveys and case series have repeatedly identified products containing lead, mercury, or arsenic, and individual case reports describe lead toxicity temporally associated with ingestion of such preparations [1,5,7]. This exposure pathway is an especially important diagnostic consideration when patients report use of complementary or alternative medicines and when routine environmental or occupational risks are absent.

Clinically, lead-related presentations are protean: vague constitutional complaints, abdominal pain (historically “saturnine colic”), constipation, neuropsychiatric symptoms, and haematologic abnormalities may all occur depending on dose and chronicity [2,3]. Because lead-induced anaemia can clinically and biochemically mimic autoimmune haemolysis or microangiopathic haemolytic anaemia (MAHA), a structured approach is required. Basic investigations should include a peripheral blood smear (with attention to stippling and schistocytes), a full haemolysis panel (reticulocyte count, LDH, bilirubin fractionation, haptoglobin), a direct antiglobulin test (to exclude immune haemolysis), and iron studies/B12/folate as indicated. In the setting of anaemia with basophilic stippling or suggestive exposure history, measurement of a venous blood lead level (BLL) is the diagnostic test of choice [8].

Management priorities are removal from exposure, supportive care, and consideration of chelation for symptomatic or sufficiently high BLLs. International and national guidance outline thresholds and clinical context for chelation; oral dimercaptosuccinic acid (succimer; DMSA) is commonly used for moderate toxicity and has established dosing regimens, whereas parenteral chelators (e.g., EDTA ± dimercaprol) are reserved for severe or encephalopathic cases and should be managed with toxicology input [8,9]. Chelation reduces circulating lead and ameliorates many acute manifestations, but clinical recovery also depends on elimination of the exposure source and supportive measures [8-10].

This case highlights three practical points for clinicians. First, an exhaustive medication and supplement history, including traditional, imported, or over-the-counter preparations, is essential when evaluating unexplained anaemia or abdominal pain [1,5]. Second, the peripheral smear remains a high-yield diagnostic tool: basophilic stippling, when present, should trigger urgent consideration of toxic exposures and directed testing [6]. Third, liaison with local toxicology/poisons services and public-health authorities is important both for individual patient management (chelation decisions, follow-up BLL monitoring) and for community risk mitigation when contaminated products are implicated [8,9].

## Conclusions

This case highlights the diagnostic complexity of lead toxicity, particularly when it presents with features suggestive of alternative aetiologies such as autoimmune haemolytic anaemia. While lead poisoning was ultimately identified, the diagnosis was delayed in part due to missed early clues, namely, basophilic stippling and ring sideroblasts, which were recognised only in retrospective review. This underscores not only the diagnostic value of peripheral smear examination and exposure history but also the potential for initial oversight. Rather than suggesting these elements are routinely sufficient for early diagnosis, this case illustrates the need for heightened clinical suspicion and systematic re-evaluation in cases of unexplained anaemia. Overall, this case offers valuable insight into the pitfalls and learning points surrounding the diagnostic journey in lead toxicity and emphasises the importance of considering environmental exposures even when initial findings are ambiguous.

## Figures and Tables

**Figure 4:**
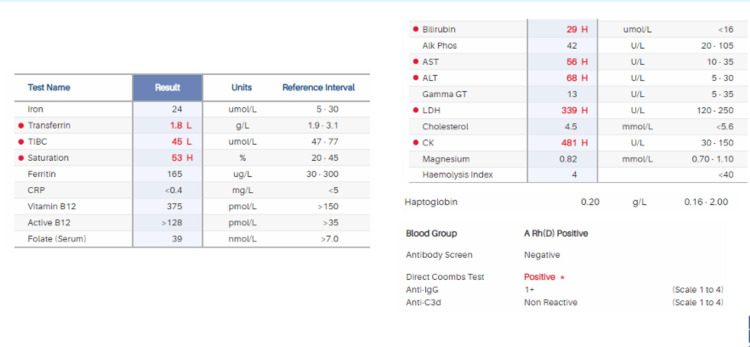
Biochemical and haematological investigations at presentation Figure 4 shows laboratory findings demonstrating anaemia with appropriate marrow response and mild biochemical evidence of haemolysis. Elevated LDH (339 U/L), indirect hyperbilirubinaemia (29 µmol/L), and low-normal haptoglobin (0.20 g/L) were noted. Direct Coombs test was weakly positive (Anti-IgG 1+, Anti-C3d non-reactive), raising suspicion for autoimmune haemolytic anaemia. Iron studies showed normal ferritin with low transferrin and total iron-binding capacity (TIBC), but increased saturation. Folate and B12 levels were within normal range. These findings contributed to diagnostic uncertainty and prompted further evaluation.

**Figure 5:**
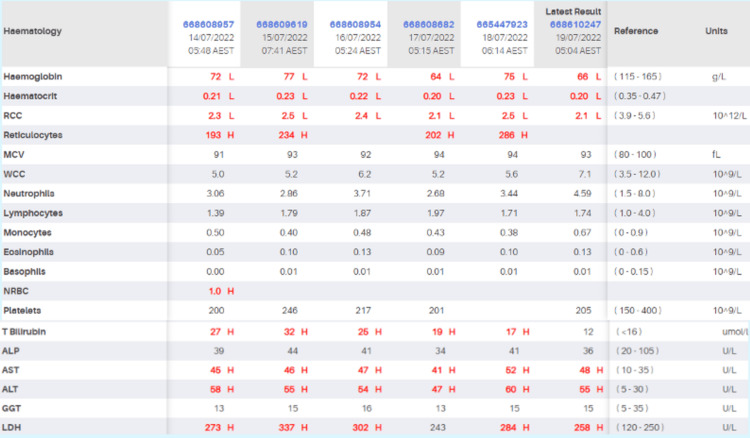
Serial haematological and biochemical parameters during admission Figure 5 shoes the trend of haematological indices over six consecutive days, showing persistent normocytic anaemia (lowest Hb 64 g/L on 17/07/2022) with sustained reticulocytosis (peak 286 × 10⁹/L), indicating ongoing marrow compensation. Red cell count (RCC) and haematocrit remained low throughout. Total bilirubin, LDH, and transaminases were variably elevated, supporting a haemolytic process, though not definitive for autoimmune haemolysis. Notably, no clear spontaneous improvement was observed until diagnosis and chelation therapy were initiated. These findings illustrate the biochemical ambiguity of the case and reinforce the diagnostic challenge prior to identifying lead toxicity.

**Figure 6:**
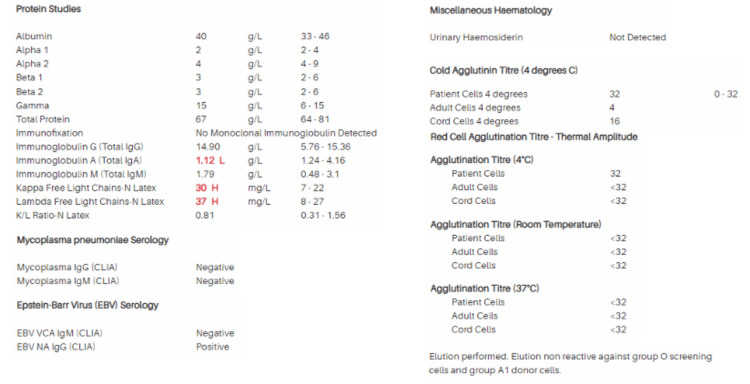
Immunological, infectious, and cold agglutinin workup Figure 6 shows expanded investigations undertaken to evaluate unexplained haemolytic anaemia. Serum protein electrophoresis and immunofixation revealed no monoclonal gammopathy. While immunoglobulin A was slightly reduced and free light chains (kappa and lambda) elevated, the kappa/lambda ratio remained normal. Infectious serologies for *Mycoplasma pneumoniae* and Epstein-Barr virus (EBV) showed negative IgM titres, with EBV NA IgG positivity consistent with past infection. Cold agglutinin testing demonstrated borderline elevation in patient cell agglutination at 4°C (titre 32; upper limit of normal), but no significant agglutination at higher temperatures, suggesting low thermal amplitude and limited clinical relevance. Urinary haemosiderin was not detected. Overall, these findings did not support an autoimmune or infectious haemolytic process, prompting reconsideration of alternative causes.
